# Editorial: The interplay of stress, health, and well-being: unraveling the psychological and physiological processes

**DOI:** 10.3389/fpsyg.2024.1471084

**Published:** 2024-08-26

**Authors:** Adelinda Araújo Candeias, Edgar Galindo, Konrad Reschke, Mariola Bidzan, Marcus Stueck

**Affiliations:** ^1^Department of Medical Sciences and Health, University of Evora, Évora, Portugal; ^2^School of Health and Human Development, University of Évora, Évora, Portugal; ^3^Comprehensive Health Research Centre, University of Evora, Évora, Portugal; ^4^Leipzig University, Leipzig, Germany; ^5^University of Gdansk, Gdansk, Poland; ^6^DPFA Akademie für Arbeit und Gesundheit, Leipzig, Germany

**Keywords:** health, stress, wellbeing, psychological processes, physiological processes

## Introduction

The World Health Organization (WHO) has identified stress as one of the foremost health crises of the 21st century. This recognition underscores the widespread impact of stress on contemporary life. In our increasingly fast-paced society, elevated levels of anxiety and dissatisfaction are fueling a stress crisis. This phenomenon is becoming the “new normal,” resulting in reduced productivity and a range of health problems. Initially, these health issues may be subtle, but over time, they can escalate into serious conditions such as hypertension, diabetes, arthritis, and inflammatory disorders, all of which are intensified by persistent stress.

This Research Topic represents the state-of-the-art research on the interplay between stress, health, and wellbeing across Europe, Asia, North America, and South America. It presents a comprehensive examination of the intricate relationships between stress, health, and wellbeing. By exploring the underlying psychological and physiological processes, this Research Topic aims to shed light on how these factors interact and influence each other, providing valuable insights into potential interventions and strategies for promoting optimal mental and physical wellbeing. It addresses key issues related to how stress impacts both physical and mental health, and explores effective intervention and prevention strategies. This Research Topic of studies provides a comprehensive examination of the social, emotional, and cognitive factors that influence wellbeing, highlighting innovative approaches and evidence-based practices to enhance resilience and health worldwide.

The exploration of the intricate relationship between stress, health, and wellbeing has become increasingly relevant in contemporary discourse, particularly in light of the global challenges posed by health crises, social upheaval, and environmental changes. This editorial aims to provide a conceptual introduction to the multifaceted interactions between these domains, drawing on the latest research findings that highlight the psychological and physiological processes involved.

The interplay of stress, physiology, and health is profound, as chronic stress has been associated with a variety of psychological mechanisms, e.g., anxiety coping styles or cognitive evaluation processes (Candeias et al., 2021; Stueck, 2021) as well as to physical health issues, including cardiovascular diseases, immune dysfunction, and metabolic disorders (Goldstein et al., 2021; James et al., 2023).

Research indicates that stress-related behaviors, such as poor sleep and unhealthy eating, exacerbate these health problems, necessitating effective intervention strategies to mitigate their impact (Galindo et al., 2022). Understanding these physiological pathways and the possibilities of intervention is crucial for developing comprehensive health promotion strategies that address both mental and physical wellbeing in individuals.

The importance of understanding the relationship between stress, health, and wellbeing has never been more critical, as societies globally strive to improve quality of life and reduce health disparities. This volume reflects the increasing interest in studying the interventions and prevention strategies that can mitigate the adverse effects of stress. By bringing together research from diverse geographical and cultural contexts, this Research Topic offers valuable insights into both universal and context-specific aspects of health and wellbeing.

The goal of this volume is to offer a multifaceted perspective on the complex interactions between stress, health, and wellbeing. By featuring a diverse array of theoretical perspectives and empirical research, the Research Topic emphasizes the importance of interdisciplinary collaboration and a holistic understanding of these interconnected domains.

This Research Topic is organized into five main key thematic categories, each exploring a different facet of the stress-health-wellbeing relationship:

1. The impact of chronic stress on physical health

In this section, the book explore the physiological processes through which chronic stress contributes to physical health problems such as cardiovascular diseases, immune dysregulation, and metabolic disorders. The articles investigate the role of stress-related behaviors, such as poor sleep, unhealthy eating habits, and a sedentary lifestyle, in mediating the relationship between stress and physical health outcomes. The research also discusses potential interventions and preventive measures to mitigate the adverse effects of chronic stress on physical wellbeing. Auer et al. examine how workplace appreciation can influence cardiovascular health, highlighting the physiological impact of stress on physical health outcomes. Aungle and Langer explore the borderline effect for diabetes: when no difference makes a difference, a research exploring the subtle impacts of stress on the management and perception of diabetes, illustrating how chronic stress can exacerbate metabolic disorders. Sabblah et al. present the assessment of occupational stress among certified registered anaesthetist's in the Greater Accra region, a research focalized on the impact of occupational stress on physical and mental health, emphasizing the physiological consequences of chronic stress. Lv et al. devote their attention to the effects of negative emotions on chronic stress and fatigue. Njoroge et al. write on the impact of stressors like the recent pandemic in the mental health of American families.

2. Psychological resilience and mental health

This section analyses the protective role of psychological resilience in buffering the negative impact of stress on mental health outcomes. Articles in this category explore the factors that contribute to the development and enhancement of resilience, such as positive emotions, cognitive flexibility, and social support networks. The articles evaluate evidence-based interventions and strategies aimed at promoting resilience and fostering mental wellbeing in the face of stressors. Kim et al. write about meaning-making while staying connected matters in psychological adaptation during the pandemic: a longitudinal moderated mediation study, investigating how meaning-making and social connections contribute to psychological resilience during the pandemic. Predko et al. examine the role of psychological hardiness in mental health resilience among individuals facing war-related stress, in a research entitled “*Psychological characteristics of the relationship between mental health and hardiness of Ukrainians during the war*”. And Hsieh et al. explore how age and resilience influence the relationship between adversity and wellbeing, highlighting protective psychological factors. Li and Meng analyze the beneficial effects of job satisfaction on the health of medical workers. On the other hand, other authors explore the role of negative emotions. Gu et al. study how fear, affect the recovery of cancer patients, and Sowan and Kissane how demoralization and intolerance of uncertainty affect the wellbeing of patients with cardiac disease.

3. Stress, wellbeing, and positive psychology

This section is devoted to the relationship between stress and subjective wellbeing, considering both hedonic wellbeing (e.g., life satisfaction, positive emotions) and eudaimonic wellbeing (e.g., sense of purpose, personal growth). The articles explore the role of positive psychology interventions, such as gratitude exercises and mindfulness practices, in enhancing wellbeing and resilience, even in the presence of stress. The research also examines the potential long-term benefits of cultivating wellbeing as a protective factor against stress-related health problems, specifically, how mindfulness interventions can enhance wellbeing and reduce negative emotions through increased resilience. Shi et al. observe the effects of nature-based therapy on mental health outcomes, focusing on mindfulness as a mediating factor. Kang et al. propose a natural method to deal with depression based on mindfulness and peace of mind. Sun et al. study the relationship between pregnancy stress and mental health. Akbulut and Erci examine the role of individual mindfulness practices in managing stress and improving health outcomes in hemodialysis patients. Liang et al. want to see how mindfulness can help to ameliorate the suboptimal health status. Ma et al. compare and evaluate various methods of regulating anxiety emphasizing the role of self-relaxation. Last, but not least, Li et al. devote their attention to the good effects of physical activity in the combat against subclinical depression.

4. The role of social support and community

This section highlights the importance of social support systems in moderating the effects of stress on health and wellbeing. Articles in this category investigate the impact of social isolation and loneliness on stress-related health outcomes and wellbeing. The research also explores community-based interventions and initiatives that promote social connectedness and resilience in the face of stress. Roland et al. examine the role of social support in mitigating the effects of pandemic-related stress on maternal health. Tolera et al. explore the impact of social and community support on the wellbeing of health service providers facing workplace violence. Guo et al. investigate community-based horticultural activities and their effects on stress reduction and mental wellbeing in children.

5. Individual differences and contextual factors

This section examines the influence of individual differences, such as personality traits and genetic predispositions, and contextual factors, such as socioeconomic status and cultural norms, on the stress-health-wellbeing relationship. The articles consider how these factors interact and shape individuals' responses to stress and their subsequent health and wellbeing outcomes. The research discusses implications for personalized interventions and targeted approaches in stress management and wellbeing enhancement. Wu et al. explore how individual differences and contextual factors influence employment stress and mental health outcomes. Li et al. investigate the impact of contextual factors on job satisfaction and wellbeing among medical staff. Seiffge-Krenke and Sattel, on their hand, explore the influence of factors like personality, self identity and parental rearing styles influence the onset of somatic complaints.

## Conclusion

The exploration of the interplay between stress, health, and wellbeing reveals a complex and multifaceted relationship that profoundly affects individuals and communities across the globe. This volume highlights how chronic stress contributes to various physical health problems, including cardiovascular diseases, immune dysfunction, and metabolic disorders. Stress-related behaviors such as poor sleep and unhealthy eating habits further exacerbate these conditions, emphasizing the need for effective interventions and preventive measures to mitigate the adverse effects of stress on physical health.

Psychological resilience emerges as a crucial protective factor in buffering the negative impacts of stress on mental health. By fostering positive emotions, cognitive flexibility, and robust social support networks, individuals can enhance their resilience and improve their mental wellbeing, even amidst significant stressors. The research underscores the importance of meaning-making, social connections, and psychological hardiness in promoting resilience, particularly during challenging times like the COVID-19 pandemic.

Positive psychology offers promising strategies for enhancing wellbeing and resilience, with interventions such as gratitude exercises and mindfulness practices demonstrating long-term benefits in reducing stress-related health problems. These approaches, along with physical activity and nature-based therapies, play a vital role in improving mental health outcomes and managing stress effectively.

The importance of social support systems is also evident in their ability to moderate the effects of stress on health and wellbeing. Community-based interventions that promote social connectedness and resilience can significantly reduce the detrimental impact of social isolation and loneliness. The research highlights how social support can alleviate stress, particularly during crises, and how community engagement contributes to stress reduction and overall mental wellbeing.

Finally, individual differences and contextual factors, such as personality traits, genetic predispositions, socioeconomic status, and cultural norms, play a significant role in shaping responses to stress and subsequent health outcomes. This volume emphasizes the need for personalized interventions and targeted approaches in stress management and wellbeing enhancement, recognizing that effective strategies must consider the diverse factors influencing stress and health.

In summary, this Research Topic of research provides a comprehensive understanding of the intricate interactions between stress, health, and wellbeing. The insights gained from these studies can provide a basis for developing effective interventions and strategies to promote optimal health, wellbeing, and resilience in the face of stressors. By integrating insights from various disciplines and cultural contexts, the volume offers valuable guidance for developing innovative and evidence-based practices to enhance resilience, reduce health disparities, and improve quality of life worldwide. As societies continue to navigate the challenges posed by stress, these findings underscore the urgent need for holistic and collaborative approaches to promoting mental and physical wellbeing. We hope this Research Topic serves as a valuable resource for researchers, practitioners, and policymakers dedicated to enhance wellbeing and to reduce the adverse effects of stress.
